# Guiding principles and proposed classification system for the responsible adoption of artificial intelligence in scientific writing in medicine

**DOI:** 10.3389/frai.2023.1283353

**Published:** 2023-11-16

**Authors:** Brett N. Hryciw, Andrew J. E. Seely, Kwadwo Kyeremanteng

**Affiliations:** ^1^Division of Critical Care, Department of Medicine, University of Ottawa, Ottawa, ON, Canada; ^2^Division of Thoracic Surgery, Department of Surgery, The Ottawa Hospital, Ottawa, ON, Canada; ^3^Clinical Epidemiology, Ottawa Hospital Research Institute, University of Ottawa, Ottawa, ON, Canada; ^4^Institute du Savoir Montfort, Ottawa, ON, Canada

**Keywords:** artificial intelligence, medicine, scientific writing, large language model, ethics, natural language processing, guidelines and recommendations, innovation

## Abstract

The integration of large language models (LLMs) and artificial intelligence (AI) into scientific writing, especially in medical literature, presents both unprecedented opportunities and inherent challenges. This manuscript evaluates the transformative potential of LLMs for the synthesis of information, linguistic enhancements, and global knowledge dissemination. At the same time, it raises concerns about unintentional plagiarism, the risk of misinformation, data biases, and an over-reliance on AI. To address these, we propose governing principles for AI adoption that ensure integrity, transparency, validity, and accountability. Additionally, guidelines for reporting AI involvement in manuscript development are delineated, and a classification system to specify the level of AI assistance is introduced. This approach uniquely addresses the challenges of AI in scientific writing, emphasizing transparency in authorship, qualification of AI involvement, and ethical considerations. Concerns regarding access equity, potential biases in AI-generated content, authorship dynamics, and accountability are also explored, emphasizing the human author’s continued responsibility. Recommendations are made for fostering collaboration between AI developers, researchers, and journal editors and for emphasizing the importance of AI’s responsible use in academic writing. Regular evaluations of AI’s impact on the quality and biases of medical manuscripts are also advocated. As we navigate the expanding realm of AI in scientific discourse, it is crucial to maintain the human element of creativity, ethics, and oversight, ensuring that the integrity of scientific literature remains uncompromised.

## Introduction

### Artificial intelligence and large language models

The advancement of artificial intelligence (AI), specifically large language model (LLM) tools, such as OpenAI’s GPT-4 or Google’s Bard, is pushing boundaries and redefining interactions within academic domains, including scientific writing (Hryciw et al., 2023; Milano et al., 2023). These LLMs are intricately designed to learn from voluminous text data, enabling the generation of contextually relevant, human-like text. They represent a tool within the broader discipline of natural language processing (NLP), which is aimed at allowing computers to understand, interpret, and generate human language (de Angelis et al., 2023). This technological breakthrough fosters an unprecedented level of human–computer interaction, bridging the gap between the intricacies of human language and the precise operations of computational algorithms.

### Opportunities for LLMs in scientific writing

The potential for LLMs in medical writing stems from their capacity to rapidly assimilate and analyze vast amounts of data, thereby facilitating the generation of insights and content at unprecedented scales. This may eventually allow for real-time synthesis of new research findings and, potentially, more timely updates in medical literature. Therefore, with the integration of AI and LLMs into the academic landscape, a wealth of opportunities is opened. Furthermore, AI functions as an unwavering academic partner, with the potential to streamline the writing process, enhance the clarity of complex ideas, and provide informed recommendations on linguistic intricacies (King, 2023). Furthermore, AI, with its proofreading capabilities, meticulously corrects minor grammar and syntax errors, promoting consistency and precision in language (Mallio et al., 2023). Finally, by transcending language barriers, AI will bolster the global exchange of knowledge, augmenting the reach and impact of scientific literature.

### Potential concerns of incorporating LLMs in scientific writing

While the integration of LLMs presents exciting prospects in scientific writing, particularly within the medical literature, it brings forth several concerns (Hammad, 2023; Hosseini et al., 2023). The first is the risk of unintentional plagiarism; LLMs generate content based on their training data and may inadvertently reproduce elements of it. In an environment where originality is paramount, such issues could undermine the credibility of the work produced. Second, medical literature often includes nuanced and context-specific information, such as clinical trial results, patient outcomes, and disease descriptions. Misinterpretations or inaccuracies by LLMs in understanding these contexts can lead to misinformation, potentially affecting patient care and scientific understanding of medical conditions. Beyond inaccuracies, there are serious ethical concerns when misinformation propagates through medical literature. Any misrepresentation or misunderstanding could lead to harmful medical practices, reinforcing the need for rigorous validation (Fournier-Tombs and McHardy, 2023). Moreover, biases inherent in the training data could influence the generated content, thus perpetuating historical biases in subsequent publications. For example, if the training data have an under-representation of certain population groups or diseases, the content of AI could unintentionally skew the narrative or focus of new medical literature. Finally, the disruptive nature of AI in scientific writing also brings forth concerns about over-reliance. There is an inherent danger that researchers might depend too heavily on AI tools, diminishing the human analytical touch that is pivotal to authentic and critical scientific discourse. It is for these reasons that, for the foreseeable future, the role of the human author alongside the AI contributor will remain crucial to the development of scientific writing, particularly for medical literature. The following guidelines put forth suggestions to provide direction and generate discussion as we welcome the dawn of the AI author.

## Governing principles of AI adoption

To responsibly navigate the integration of AI in scientific writing for medical literature, we must consider and adhere to a set of fundamental principles that ensure integrity, transparency, validity, and accountability.

- *Integrity*: Maintaining ethical boundaries and accuracy in AI-assisted content is paramount. AI should serve as an aid to human effort, rather than a replacement, upholding the ethical standards intrinsic to scientific publishing.
- *Transparency*: Clear disclosure of AI involvement, including its identity, and the extent of human oversight are essential for accountability in the AI-augmented manuscript development process.
- *Validity*: Ensuring factual and accurate AI-generated content is a necessity. Rigorous human oversight in checking references, identifying biases, and verifying facts ensures the validity of the final manuscript.
- *Accountability*: As human authors, the ultimate responsibility for the final manuscript rests on our shoulders. We should be prepared to justify the content of the manuscript and ensure that it meets the rigorous ethical and quality standards established within the scientific publishing community.

Navigating this AI-integrated landscape calls for a delicate balance of harnessing the potential of AI, recognizing its limitations, and adhering to these guiding principles of integrity, transparency, validity, and accountability. In doing so, we can augment our scientific pursuits with AI, maintain our commitment to academic integrity, and continue our noble pursuit of knowledge in the realm of scientific publishing.

## Guidelines for reporting AI involvement

To maintain principles in scientific writing, we propose the following approach for reporting AI involvement in medical manuscript development:

- *Specify the level of AI assistance*: Clearly state the extent of AI involvement, ranging from proofreading to, perhaps eventually, full manuscript generation.
- *Detail human involvement and oversight*: Specify the degree of human involvement and oversight throughout the manuscript development process, including editing, revising, and validating AI-generated content.
- *Describe the AI tool or model*: Provide a brief description of the AI tool or model utilized, including the software name and version number where appropriate, the LLM or AI tool, and primary functions.
- *Address ethical considerations*: Disclose any ethical considerations related to the use of AI in manuscript development, such as potential biases relevant to the subject matter.
- *Acknowledge limitations*: Recognize the limitations of AI use, including potential biases, inaccuracies, or misinterpretations that may arise from AI-generated content.
- *Disclose conflicts of interest*: Reveal any potential conflicts of interest, particularly when using commercial AI tools, to maintain transparency and avoid potential biases.

## Classification system for AI involvement

In considering the classification of AI involvement in medical manuscript development, we propose an intuitive system to specify the level of AI assistance that aligns with the real-world applications of AI in academic writing and provides an accurate representation of the spectrum of contributions of AI to the academic field. We propose that the level of AI involvement is classified based on the highest level of usage as follows:

- *Proofreading*: At this initial level of involvement, the human author is exclusively responsible for the written content. AI tools are used to proofread, ensure grammatical accuracy, and correct syntactical errors.
- *Restructuring*: This involves the use of AI tools for rewording, paraphrasing, and reorganizing existing content. However, at this level, AI can also be involved in idea or content generation that is extensively revised by authors, leaving the initial output only minimally recognizable.
- *Drafting*: This category represents a higher level of AI involvement, where tools such as GPT-4 are used to generate a working draft based on input data and human guidance. The AI-generated content here would be expected to require notable revisions by human authors, resulting in distinct differences from the original AI output.

Although we do not feel that the current state of AI technology permits entirely autonomous manuscript generation with negligible human oversight, we anticipate that future advancements in AI could make this possible. Consequently, we foresee a fourth category, which we provisionally term “**Autonomous Writing**.” In this hypothetical category, AI could have the capacity to autonomously generate a complete manuscript requiring only minimal human editing and oversight. However, it is crucial to approach this future category with caution. The proposition of AI producing manuscripts autonomously underscores the importance of maintaining human oversight and accountability in the process. As AI continues to evolve, human involvement and the governing principles of AI adoption remain central to ensuring ethical academic practices and preserving the integrity of scientific literature.

## Comparison with existing guidelines

While our guidelines share common elements with existing AI ethics frameworks and human–AI collaboration guidelines, they bring additional focus to the unique challenges of AI in scientific writing, particularly in the medical domain. For instance, where traditional AI ethics guidelines emphasize avoiding harm and maintaining transparency in a broad sense (Floridi and Cowls, 2019; Raja and Havens, 2019), our proposed guidelines delve into the specifics of transparency in authorship, the qualifying the extent of AI involvement, and how to mitigate some of the ethical nuances of AI-generated content for medical literature, including guidance on how to ensure accuracy and validity of AI-generated content while minimizing and acknowledging biases. Existing explorations of human–AI collaborations lay a foundation for AI-integration but do not directly address a standard for disclosing the role of AI in creating academic content or a specific method for reporting the extent of involvement (Salvagno et al., 2023), which is a key aspect of our guidelines. Our guidelines, thus, represent a first step toward a comprehensive framework that builds upon existing literature but also continues to address the unique challenges posed by using AI in medical manuscript development.

## Ethical considerations

Ethical considerations surrounding the use of AI and LLMs in medical manuscript development could be categorized into several domains, including access equity, data bias, authorship, and accountability. First, in terms of access equity, the use of AI and LLMs in scientific writing could lead to widening a gap between well-resourced institutions that can afford these advanced tools and those with fewer resources. This raises concerns about the potential for unequal representation in scientific discourse and could affect the breadth and diversity of research outputs. Since AI-generated content is based on a sample of training data, it is prone to the biases present in this data. Therefore, if certain populations, conditions, or perspectives are under-represented in the training data, these will also be under-represented in the AI-generated content, potentially further skewing the representation in the scientific literature. Together, this interaction between access equity and data bias could further influence the literature to overrepresent well-resourced populations. This highlights the need to ensure that AI training data are broadly representative in an effort to maintain equity for all patients, especially those who represent vulnerable or marginalized populations (Zou and Schiebinger, 2018; Mehrabi et al., 2019). Authorship in the context of AI-assisted manuscripts raises important ethical considerations as well; however, it is the transparency surrounding its use that is of primary importance. While AI tools can contribute significantly to a manuscript, authorship may not be strictly necessary. Instead, a clear and comprehensive declaration of AI involvement, along with detailed descriptions of its usage, should be provided as per the proposed guidelines. Finally, accountability is a significant issue. As human authors, we must take responsibility for the content produced with the help of AI and be prepared to verify and stand by the information presented, ensuring that it meets the ethical and quality standards of scientific publishing. AI-based decisions, while based on data, lack the moral reasoning and intuition inherent in humans. Thus, even in the case of an unintended harmful outcome due to the content of AI, human authors should remain at the forefront of responsibility. While this is, by no means, an exhaustive discussion of the potential ethical considerations, it serves to generate discussion and highlight some of the important ethical considerations of AI adoption in the scientific writing of medical literature.

## Recommendations for future research and policy development

As AI and NLP continue to reshape our technological landscape, and by extension, scientific writing in medical literature, we must proactively prepare for this reality. We endorse the following actions: (i) foster collaboration between AI developers, researchers, and journal editors to create AI tools and guidelines that satisfy the needs of all stakeholders while maintaining the quality of scientific writing; (ii) promote education and training for researchers on the responsible use of AI in academic writing, emphasizing the importance of critical thinking and human oversight; and (iii) regularly evaluate the impact of AI on the quality and biases of medical manuscripts as technology and its integration continue to evolve.

## Conclusion

As we venture into an era where AI-generated content meets or even surpasses human-level quality, it becomes critical to establish robust guidelines and a classification system reflecting the role of AI accurately. Our proposed approach and classification system empower researchers to unlock the full potential of AI technology while addressing potential ethical and quality concerns. As we recognize and harness the strengths of AI, we must stay vigilant to ensure that the human element of creativity, ethics, and oversight is maintained, preserving the integrity of scientific literature. Moreover, as AI continues to disrupt the medical field, we must keep evolving our ethical guidelines to remain in lockstep with the rapidly changing technology landscape. Staying updated ensures that while we gain from AI’s capabilities, we also remain guardians of ethical practices in medical research and literature.

## Declaration

- In the development of this article, we employed AI tools for article drafting.
- The AI-produced content from structured and increasingly tailored input data underwent significant revisions by the authors to ensure accuracy and reliability. We maintained constant human oversight throughout the process, including editing, revising, and validating the AI-generated content.
- GPT-4, a Large Language Model developed by OpenAI, was used through ChatGPT August 3 Version and Perplexity. This software has the primary function of generating human-like text based on input prompts, demonstrating an ability to understand and produce contextually relevant content.
- We acknowledge potential ethical (and ironic) concerns about using AI-generated content to generate recommendations regarding AI involvement in medical writing and therefore took care to re-evaluate and edit the output created.
- We acknowledge potential inaccuracies or misinterpretations that may arise from AI-generated content and therefore took care to verify the output.
- No conflicts of interest were present in the use of the AI model for this article.

## Data availability statement

The original contributions presented in the study are included in the article/supplementary material, further inquiries can be directed to the corresponding author.

## Author contributions

BH: Conceptualization, Writing – original draft, Writing – review & editing. AS: Writing – review & editing. KK: Conceptualization, Writing – review & editing.
